# Long Tube Feeding Bottles

**Published:** 1899-07-01

**Authors:** 


### LONG TUBE FEEDING BOTTLES.

At the recent meeting of the American Medical Association, held at Columbus, Ohio, Dr. Ernest Wende read a paper in which he showed by bacteriological experiment how impossible it is to clean the long tube feeding bottle which has so long dominated the nurseries in so many lands, much to the detriment of hand-fed infants in all parts of the world. The mischief with these bottles is not merely that the inner surface of the tubes get dirty and are difficult to clean, but that the rubber itself becomes affected by the microbes, which eat into it so that cavities occur within its substance, thus rendering cleansing quite impossible. Tubes which had been used, when left in an incubator for 20 days, became disintegrated by the action of the infecting bacilli, and in some cases became actually liquefied. It was stated in the discussion that the same thing held good in regard to rubber nipples, the unevenness of surface caused by the action of the microbes, and the growth of these organisms within the rubber, making it quite impossible to maintain bacteriological cleanliness. We gather, also, from the discussion that in certain cities in the States the use of the long tube has been prohibited, and that this prohibition has been attended with the best possible effect upon the infant mortality.

